# A Giant Gastroschisis Associated with Pulmonary Hypoplasia and Spinal Anomaly: A Case Report and a Literature Review

**DOI:** 10.1155/2018/8378769

**Published:** 2018-05-02

**Authors:** Surasak Puvabanditsin, Robin Burger, Vidya Puthenpura, Lauren Walzer, Adaora Madubuko, Christine Minerowicz, Rajeev Mehta

**Affiliations:** ^1^Department of Pediatrics, Rutgers Robert Wood Johnson Medical School, New Brunswick, NJ, USA; ^2^Department of Pathology, Rutgers Robert Wood Johnson Medical School, New Brunswick, NJ, USA

## Abstract

Gastroschisis most often occurs as an isolated anomaly and extragastrointestinal associations are rare. Most commonly, the anomalies associated with gastroschisis are cardiac and central nervous system abnormalities. Respiratory insufficiency has sometimes been reported in association with giant abdominal wall defects. Poor outcomes and prolonged ventilator support have been reported in giant gastroschisis and omphalocele, especially if associated with herniation of the majority of the liver. We report a case of a large gastroschisis that was associated with a kyphoscoliosis and pulmonary hypoplasia.

## 1. Introduction

Gastroschisis is a right-sided, small, and full thickness paraumbilical defect of the abdominal wall with a prevalence rate of about 4 per 10,000 live births [1]. Unlike in the case of an omphalocele, the herniated bowel is in direct contact with amniotic fluid. Theories concerning the etiology of gastroschisis are usually attributed to the result of a vascular insult. Barisic et al. 2001 reported that 14% of the fetuses with gastroschisis had additional anomalies, with central nervous system and cardiac malformations being the most common [2]. Intrauterine demise is uncommon and generally related to other major anomalies. Although the spinal anomaly has been reported in association with complex abdominal defect (e.g., body stalk anomaly), isolated gastroschisis with spinal anomaly has not been previously reported. We report rare and fatal anomalies in a preterm neonate with a large gastroschisis, severe pulmonary hypoplasia, and kyphoscoliosis.

## 2. Case Report

**Figure 1:**
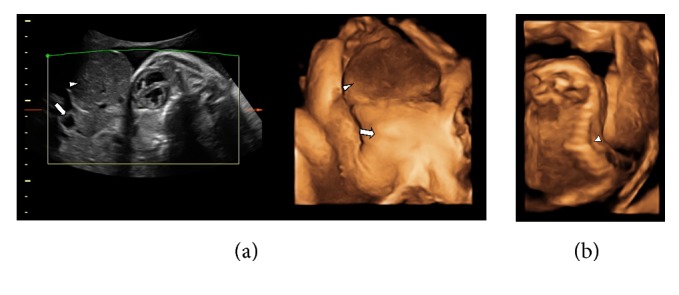
(a) 2D and 3D ultrasound image showing abdominal wall defect with liver (short arrow) and small bowel (long arrow) herniation at 33 weeks of gestation. (b) 3D ultrasound image showing kyphosis of the thoracolumbar spines at 22 weeks of gestation (arrow).

A 1950-g female infant was born at 36 weeks' gestation by cesarean section for multiple fetal anomalies to a 25-year-old G5 P2 African American woman. Apgar scores were 2, 2, and 2, at 1, 5, and 10 minutes, respectively. Amniotic fluid was meconium stained. Prenatal ultrasound at 22 weeks of gestation had revealed a large abdominal wall defect with most abdominal contents lying outside, severe kyphosis of the spine, and a small chest. A prenatal ultrasound at 31 weeks confirmed the gastroschisis and revealed pulmonary hypoplasia, deviation of the heart to the left side of the chest, and a sacral spina bifida (Figures 1(a) and 1(b)). Amniocentesis was performed. The single nucleotide polymorphism (SNP) microarray analysis was normal. Although the patient was informed about the bleak outcome of her fetus, she opted for expectant management. There was no history of in utero exposure to known teratogens. Family history was negative for congenital anomalies. Physical examination of the infant revealed a weight of 1950 g (<5th centile), length of 38 cm (5th centile), and head circumference of 32 cm (40th centile). Multiple anomalies were noted at birth including a large abdominal wall defect, scoliosis, small chest, hypertelorism, flat nasal bridge, bulbous tip nose, prominent occiput, and sacral dimples. After intubation and resuscitation in the operating room, the infant was admitted to the NICU where she died within an hour after birth.

## 3. Autopsy Findings

**Figure 2:**
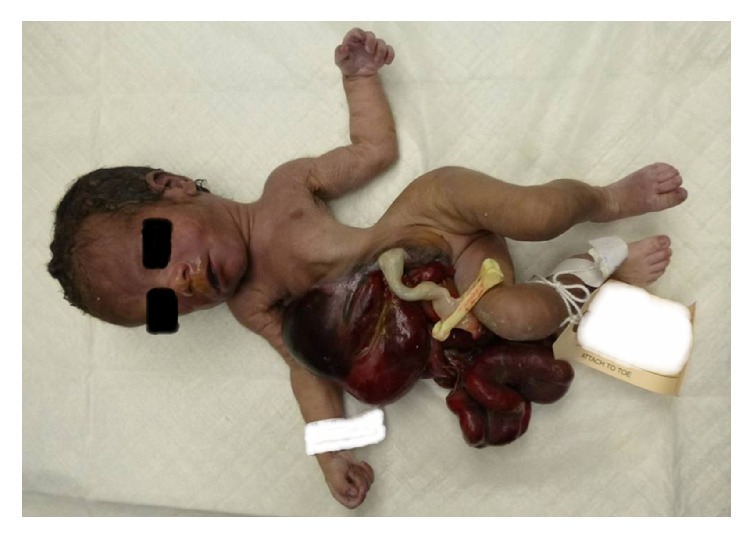
Gastroschisis is shown; note the fact that the defect is to the right of the umbilical cord and the liver and bowel are not covered by a sac. Note significantly damaged bowel with evidence of matting, foreshortening, and peel.

**Figure 3:**
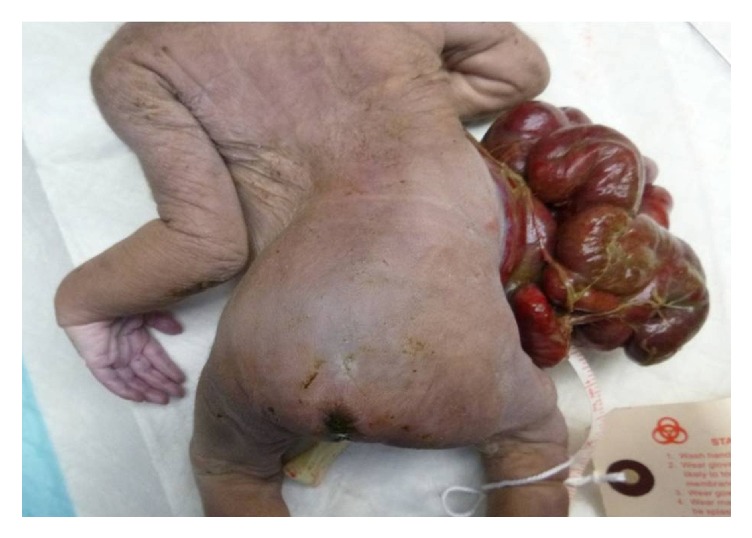
Figure shows kyphoscoliosis of the thoracolumbosacral spines and sacral dimples.

External examination showed an abdominal wall defect (9 × 7 cm in size) located to the right of the midline umbilicus. Herniation through the defect of the liver, spleen, pancreas, stomach, bowel, and uterus was noted. Fibrinous, meconium-containing exudates were evident over the serosa of the herniated abdominal organs (Figure 2). There was compression deformity of the right chest wall due to the herniated liver and abdominal organs. Additional findings included a tiny chest and sternum, severe kyphoscoliosis of the thoracolumbosacral spines, and sacral dimples with an overlying tuft of hair (Figure 3).

**Figure 4:**
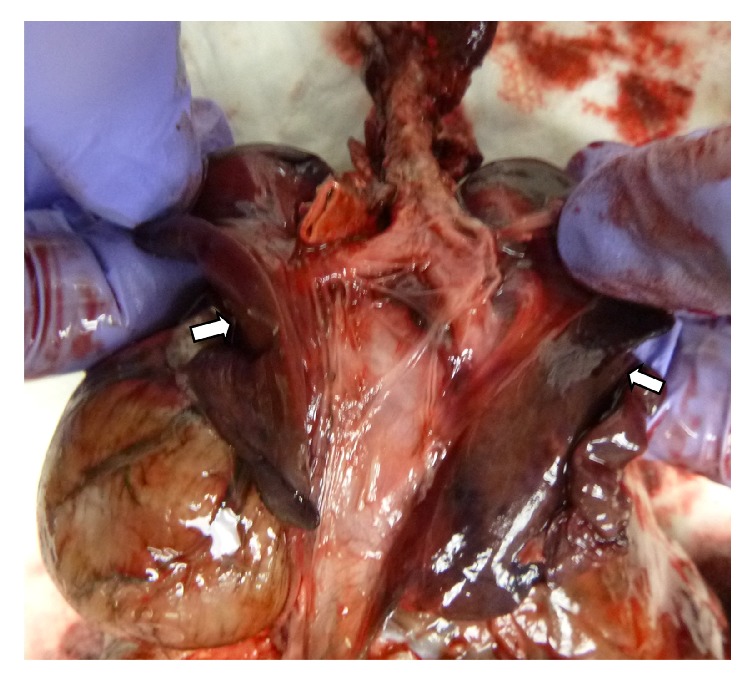
Figure shows hypoplastic lung (arrows).

**Figure 5:**
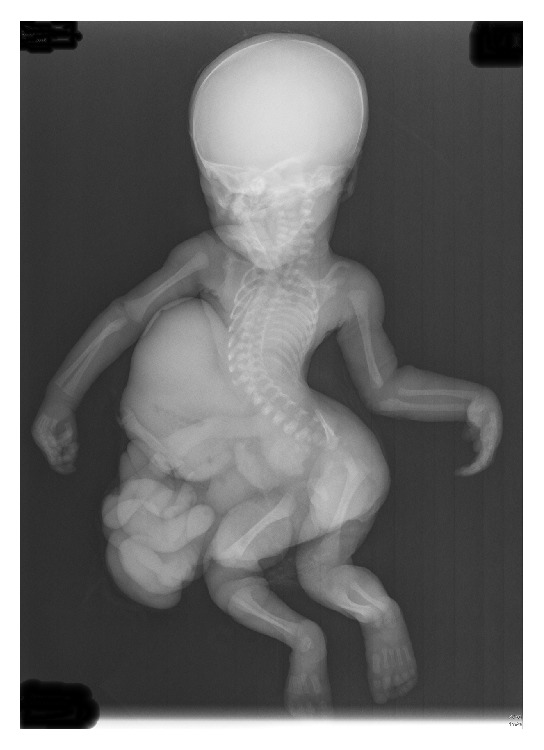
Infantogram showing a thoracic cage deformity, characterized by a narrow chest and downslanting ribs and severe kyphoscoliosis of the spines.

Internal examination revealed hypoplastic abdominal and thoracic cavities with an intact diaphragm. The heart was normal. The thoracic cage deformity was characterized by a narrow chest and downslanting ribs. There was no ossification of the sternum, and the thoracic spines protruded anteriorly into the thoracic cavity. The kidneys and adrenal glands were displaced inferiorly within the retroperitoneal space. The ovary, fallopian tubes, and uterus were normal. Both lungs were hypoplastic. The combined weight of the lungs was 9.2 grams, compared to the average weight of 45 grams. The left lung weighed 3.8 grams and the right lung weighed 5.4 grams (Figure 4). There was incomplete fusion of the sacral vertebrae. The umbilical cord and placenta were unremarkable. Postmortem infantogram showed a small and narrow chest with 12 pairs of ribs, and severe kyphoscoliosis of the thoracolumbosacral spine (Figure 5).

## 4. Discussion

Abdominal wall defects (AWDs) are a complex group of anomalies, and include the common AWDs (gastroschisis and omphalocele) as well as the complex AWDs (body stalk anomaly, abdominoschisis, pentalogy of Cantrell, bladder exstrophy, and cloacal exstrophy). The overall prevalence of AWD is 1 per 10,000 births [1]. Gastroschisis and bladder exstrophy are the only AWDs where the defect is separate from normal cord insertion. Our case had a normal umbilical cord and the insertion site was separate from the defect.

In gastroschisis, the small intestine, and occasionally the stomach or colon, is present outside of the body without a membranous protective sac. The abdominal organs protrude through a small opening that is usually to the right of the umbilicus, and the rectus muscles are entirely intact and normal [3]. Left-sided gastroschisis is extremely rare and its etiology may be different from that of right-sided gastroschisis [3]. Generally, gastroschisis is isolated [4, 5] and always includes the small intestine. However, the stomach, colon, and gonads may also be found outside the body cavity.

Over the past few decades, there is a global increase in the incidence of gastroschisis, which seems to be confined to specific demographic regions [3]. This observation, along with the defect's consistent association with factors such as young maternal age, low socioeconomic status, poor prenatal care, maternal infections, low body mass index (BMI), smoking, consumption of alcohol, and therapeutic and recreational drugs during pregnancy [6–11], suggests that environmental factors may be candidates as potential teratogens. A more recent theory suggests that gastroschisis may result from failure of one or more folds responsible for wall closure and the subsequent inhibition of the yolk stalk to merge with the connecting stalk. As development proceeds the primary intestinal loop either herniates into the amniotic cavity (instead of the umbilical cord) or a part of the intestinal loop herniates normally into the umbilical cord while another part herniates through an unclosed portion of the malformed body wall [3, 12]. Vasoconstrictive agents are often reported to be used during pregnancy by mothers of newborns with gastroschisis. However, they are unlikely to be the sole contributors to the defect. Although intestinal atresia occurs in almost 28% of infants, nonintestinal anomalies are rare.

Pulmonary hypoplasia is rarely reported in association with gastroschisis. Abnormalities of prenatal lung growth in infants with giant abdominal wall defects may be the result of an in utero deformation sequence, with several potential contributing factors [13, 14]. The displacement of the liver interferes with the molding of the lower thoracic cage. Because of the absence of the viscera, there is decreased intra-abdominal pressure with a tendency of the lower rib cage to collapse inwards with breathing movements (normally, it should moving outwards).

In many infants with giant omphalocele, the rectus abdominus muscles attach laterally to the costal margins of the ribs instead of meeting in the midline superiorly [13]. The laterally displaced abdominal muscles then exert a downward force on the rib cage, causing a more caudal declination of the ribs and narrowing of the chest wall. Together, these factors cause the developing chest to assume a narrow configuration with downslanting ribs. In our case the displacement of the liver and lateral displacement of the abdominal muscles were remarkable.

Prenatal ultrasound evaluation of fetuses with abdominal wall defects is generally able to identify pulmonary hypoplasia [12, 15]. A 2D ultrasonographic measurement of the transverse lung to thorax ratio can be used to predict the presence of pulmonary hypoplasia in fetuses with abdominal wall defects [13]. Sonographic measurement of chest/trunk length ratio can detect the narrow, elongated chest that is seen in some infants with abdominal wall defects [13]. More recently, ultrafast fetal MRI images were used to calculate total lung volumes in fetuses with giant omphaloceles [16].

Our case was prenatally initially suspected to be a body stalk anomaly because of the associated anomalies: kyphoscoliosis and pulmonary hypoplasia. There was no limb anomaly, the umbilical cord appeared normal, and the insertion site was separate from the AWD. Recognition of complex AWDs is vital for appropriate prenatal counseling and management. Our case is unusual because the large gastroschisis was associated with severe kyphoscoliosis and pulmonary hypoplasia. The spinal anomaly has not previously been reported in association with isolated gastroschisis.
